# Establishment of sex difference in circulating uric acid is associated with higher testosterone and lower sex hormone-binding globulin in adolescent boys

**DOI:** 10.1038/s41598-021-96959-4

**Published:** 2021-08-30

**Authors:** Yutang Wang, Fadi J. Charchar

**Affiliations:** 1 School of Science, Psychology and Sport, Federation University Australia, Ballarat, VIC Australia; 2 Discipline of Life Sciences, School of Science, Psychology and Sport, Federation University Australia, Ballarat, VIC 3350 Australia

**Keywords:** Medical research, Risk factors

## Abstract

Men have higher circulating levels of uric acid than women. This sex difference is suspected to be a result of suppressive effects of estradiol on uric acid. If so, estradiol would be inversely associated with circulating uric acid. This study aimed to test this hypothesis. This cross-sectional study included 9472 participants (weighted sample size of 184,342,210) aged 12–80 years from the 2013 to 2016 US National Health and Nutrition Examination Survey. Associations of sex hormones with uric acid were analyzed using weighted least squares regression, adjusting for demographic characteristics, lifestyle risk factors, and comorbidities. Neither free nor bioavailable estradiol was inversely associated with circulating uric acid in adolescent boys or girls, or adult men or women, or perimenopausal women after full adjustment. The sex difference in uric acid was established during adolescence as a result of a dramatic increase in uric acid in adolescent boys. During adolescence, the increase in estradiol in girls over time was accompanied by a relatively unchanged level of uric acid. All three fractions of estradiol (free, bioavailable, and total) were positively associated with uric acid in adolescent boys and girls after full adjustment. In adolescent boys, all three fractions of testosterone were positively associated with serum uric acid, and sex hormone-binding globulin was inversely associated with uric acid after full adjustment. These results suggest that estradiol is not inversely associated with circulating uric acid in adolescents and the establishment of sex difference in circulating uric acid during adolescence is associated with higher testosterone and lower sex hormone-binding globulin in adolescent boys.

## Introduction

Uric acid is the end product of metabolic breakdown of purine compounds^1^ which is catalyzed by xanthine oxidase^2^. Uric acid is excreted mainly via the kidney^3^. Many factors affect circulating uric acid levels, such as age, sex, ethnicity, body mass index^4–7^ as well as lifestyle factors including alcohol consumption, smoking, and physical activity^6,8,9^. In addition, kidney disease can impair the excretion of uric acid by the kidney and thus can increase uric acid levels in the circulation^6,10^. Moreover, high circulating uric acid has been reported to be associated with many health problems such as hypertension^11^, diabetes^12^, hypercholesterolemia^13^, coronary heart disease^14^, stroke^15^, sleep disorders^16^, gout^17^, and cancer^18^, which may be associated with the pro-inflammatory effect of uric acid^19^.

It is well known that men have higher circulating levels of uric acid than women^20–23^. Experts in the field suspect that this physiological difference in uric acid may be a result of estrogen^20,21^ because estradiol can inhibit the uric acid-generating enzyme xanthine oxidase isolated from rat liver^24^; in addition, estradiol replacement therapy can decrease circulating uric acid pharmacologically^25^. However, whether estradiol regulates uric acid levels under physiological conditions is unknown. If estradiol decreases uric acid levels under physiological conditions, estradiol is expected to be inversely associated with circulating uric acid in a general population. This study aimed to test this hypothesis using a representative US cohort of adolescents and adults who attended the National Health and Nutrition Examination Survey (NHANES) from 2013 to 2016.

## Results

### The characteristics of the cohort

This study included a total of 9472 noninstitutionalized US residents (weighted to a national sample size of 184,342,210) aged 12–80 years, with a mean (SD) age of 40.7 (17.9) years. Table 1 and Supplementary Table S1 describe the characteristics of the cohort. Compared to adolescents, adult participants had a higher body mass index and a lower eGFR, were less physically active, and had a higher prevalence of hypertension, diabetes, hypercholesterolemia, coronary heart disease, stroke, gout, sleep disorder, and cancer (Table 1).

**Table 1:** Characteristics of the cohort stratified by sex and age.

	Males		Females		
	Adolescent boys (12–19 years)	Adult men (20–80 years)	Adolescent girls (12–19 years)	Adult women (20–80 years)	Perimenopausal women (47–56 years)
No., unweighted	1042	4037	993	3400	589
No., weighted	13,448,725	88,248,186	12,291,504	70,353,795	12,736,818
tE, median (IQR), pg/mL	18.5 (11.6–25.5)	23.6 (18.5–29.1)	52.7 (29.7–108.0)	37.0 (8.1–100.0)	14.0 (5.4–72.2)
fE, median (IQR), pg/mL	0.4 (0.2–0.5)	0.5 (0.4–0.6)	1.0 (0.5–1.8)	0.7 (0.1–1.6)	0.3 (0.1–1.1)
bE, median (IQR), pg/mL	12.4 (7.4–17.8)	14.8 (11.5–18.7)	30.9 (16.2–58.3)	19.8 (4.2–48.4)	8.1 (3.1–33.0)
tT, median (IQR), ng/dL	378 (223–514)	396 (299–517)	24.5 (17.7–32.4)	21.4 (15.3–29.8)	19.0 (13.6–26.3)
fT, median (IQR), ng/dL	7.7 (4.5–10.3)	7.1 (5.6–9.1)	0.3 (0.2–0.5)	0.2 (0.2–0.4)	0.2 (0.2–0.3)
bT, median (IQR), ng/dL	191 (104–257)	170 (132–221)	7.6 (5.1–11.1)	5.7 (3.8–8.6)	5.0 (3.5–7.7)
SHBG, median (IQR), nmol/L	34.6 (23.8–50.1)	37.1 (26.5–52.7)	52.1 (34.8–79.9)	60.5 (40.9–90.3)	60.2 (38.5–85.9)
Uric acid, mean (SD), mg/dL	5.6 (1.2)	6.0 (1.2)	4.4 (1.0)	4.7 (1.1)	4.7 (1.1)
Age, mean (SD), years	15.4 (2.2)	45.6 (16.1)	15.3 (2.2)	43.8 (15.4)	51.3 (2.9)
BMI, median (IQR), kg/m^2^	22.3 (19.6–26.6)	28.0 (24.8–32.0)	23.0 (20.2–27.8)	27.8 (23.4–33.5)	28.5 (23.7–33.8)
eGFR, mean (SD), mL/min per 1.73 m^2^	137.2 (19.6)	96.9 (17.6)	131.6 (16.2)	101.3 (19.1)	94.5 (14.3)
**Ethnicity, %**					
Hispanic	23.7	16.2	24.6	17.5	14.4
Non-Hispanic white	54.7	64.8	52.5	61.1	64.9
Non-Hispanic black	12.4	9.9	12.9	11.7	12.3
Other	9.2	9.0	10.0	9.7	8.4
**Health status, %**					
Excellent	15.2	9.2	14.2	9.7	11.2
Very good	39.9	28.4	38.3	30.2	29.9
Good	32.0	41.0	33.9	33.9	29.3
Fair	8.4	13.9	8.3	13.8	15.1
Poor	0.7	1.7	1.4	2.0	3.0
Unknown	3.8	5.9	3.9	10.5	11.6
**Physical activity, %**					
0 min per week	17.1	42.7	25.9	44.5	43.3
1–149 min per week	9.4	14.5	14.5	17.4	19.2
150–299 min per week	11.3	12.4	15.3	13.1	14.6
≥ 300 min per week	59.9	30.3	42.0	24.9	22.9
Unknown	2.3	0.0	2.3	0.0	0.0
**Smoking status, %**					
Never	18.2	50.5	18.5	64.9	56.8
Former	0.5	28.5	0.8	17.4	21.5
Current	4.1	21.0	1.5	17.6	21.7
Unknown	77.2	0.0	79.3	0.0	0.0
**Alcohol consumption, %**					
Non-drinker	8.7	7.6	11.0	15.2	13.4
Former drinker	1.8	5.8	1.3	12.2	11.2
Current drinker	11.0	80.5	7.0	61.8	63.3
Unknown	78.5	6.1	80.7	10.7	12.0
Hypertension, %	1.4	30.2	1.0	25.4	13.4
Diabetes, %	0.5	10.0	0.5	6.9	9.1
Hypercholesterolemia, %	1.3	34.9	1.4	25.2	34.3
CHD, %	0.0	3.4	0.0	0.9	0.3
Stroke, %	0.0	2.0	0.0	1.5	2.3
Gout, %	0.0	4.5	0.0	0.9	0.6
Sleep disorder, %	5.9	24.3	5.9	28.1	34.5
Cancer, %	0.0	8.4	0.0	6.7	6.5

*bE, fE or tE* bioavailable, free or total estradiol; *BMI* body mass index; *bT, fT or tT* bioavailable, free or total testosterone; *CHD* coronary heart disease; *eGFR* estimated glomerular filtration rate; *IQR* interquartile range; *No.* number; *SD* standard deviation; *SHBG* sex hormone-binding globulin.

### Patterns of circulating levels of uric acid and sex hormones over the lifetime

**Figure 1:**
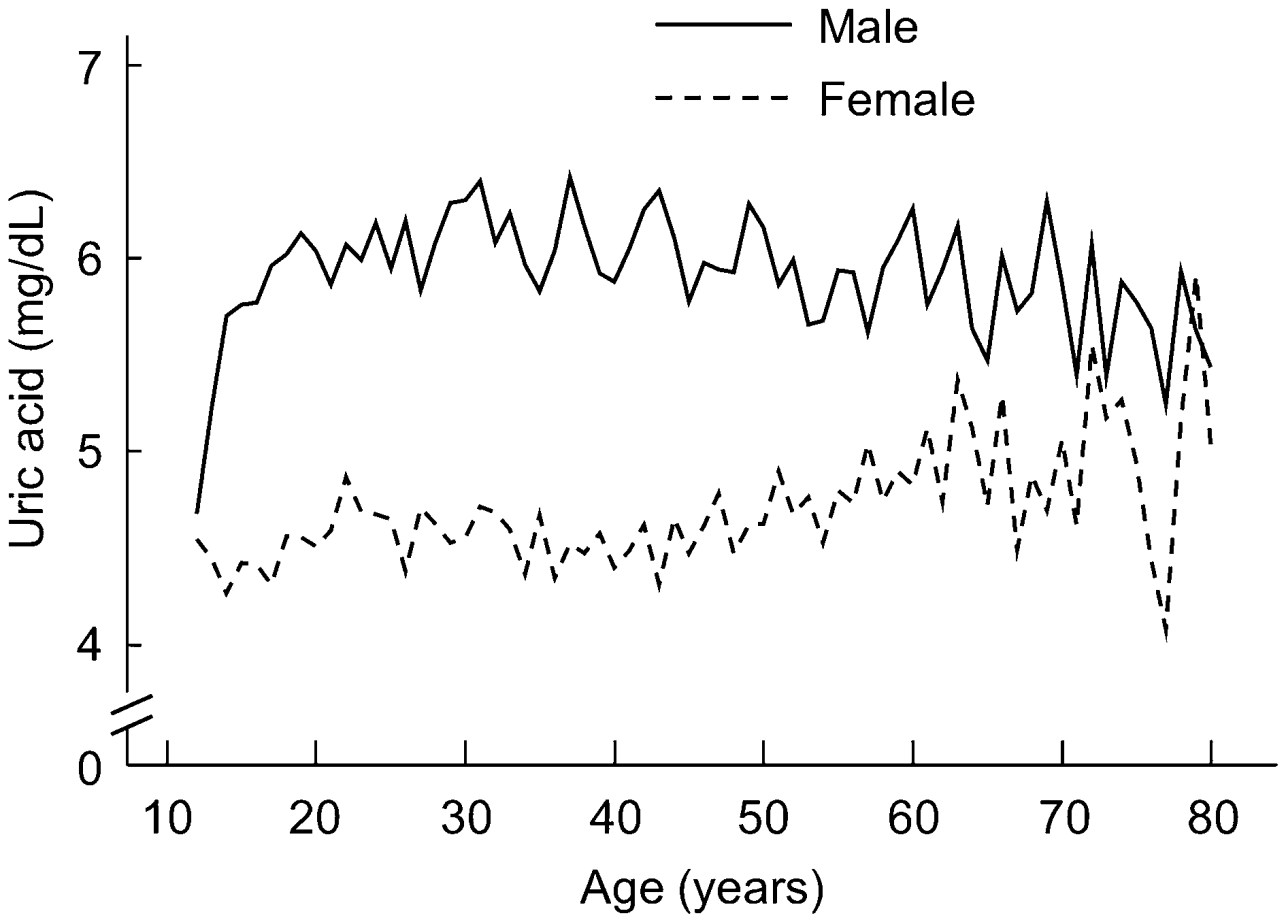
Serum levels of uric acid in males and females. This cohort included 5079 males and 4393 females, which represented a weighted sample size of 101,696,911 and 82,645,299, respectively. Data represent the weighted mean values over each year of age from 12 to 80 years.

Uric acid in males increased during adolescence (Fig. 1 and Table 2) and slightly decreased during adulthood (Fig. 1 and Table 2), whereas in females its level maintained relatively unchanged during adolescence (Fig. 1 and Table 2) and increased during adulthood (Fig. 1 and Table 2). Sex difference in circulating uric acid was established during adolescence as a result of a dramatic rise in uric acid in adolescent boys (Fig. 1).

**Table 2:** Associations of age (independent variable) with log-transformed estradiol, log-transformed testosterone, or uric acid (dependent variables) analyzed by weighted least squares regression.

	12–19 years				20–80 years			
	Males		Females		Males		Females	
	β	*P* value	β	*P* value	β	*P* value	β	*P* value
Log tE	0.619	< 0.001	0.099	0.002	− 0.002	0.912	− 0.609	< 0.001
Log fE	0.626	< 0.001	0.079	0.013	− 0.174	< 0.001	− 0.595	< 0.001
Log bE	0.636	< 0.001	0.071	0.025	− 0.237	< 0.001	− 0.598	< 0.001
Log tT	0.509	< 0.001	0.241	< 0.001	− 0.114	< 0.001	− 0.236	< 0.001
Log fT	0.577	< 0.001	0.157	< 0.001	− 0.449	< 0.001	− 0.208	< 0.001
Log bT	0.585	< 0.001	0.143	< 0.001	− 0.488	< 0.001	− 0.215	< 0.001
Log SHBG	− 0.423	< 0.001	0.035	0.268	0.434	< 0.001	0.022	0.202
Uric acid	0.317	< 0.001	0.014	0.661	− 0.092	< 0.001	0.116	< 0.001

*bE, fE or tE* bioavailable, free or total estradiol; *bT, fT or tT* bioavailable, free or total testosterone; *SHBG* sex hormone-binding globulin.

**Figure 2:**
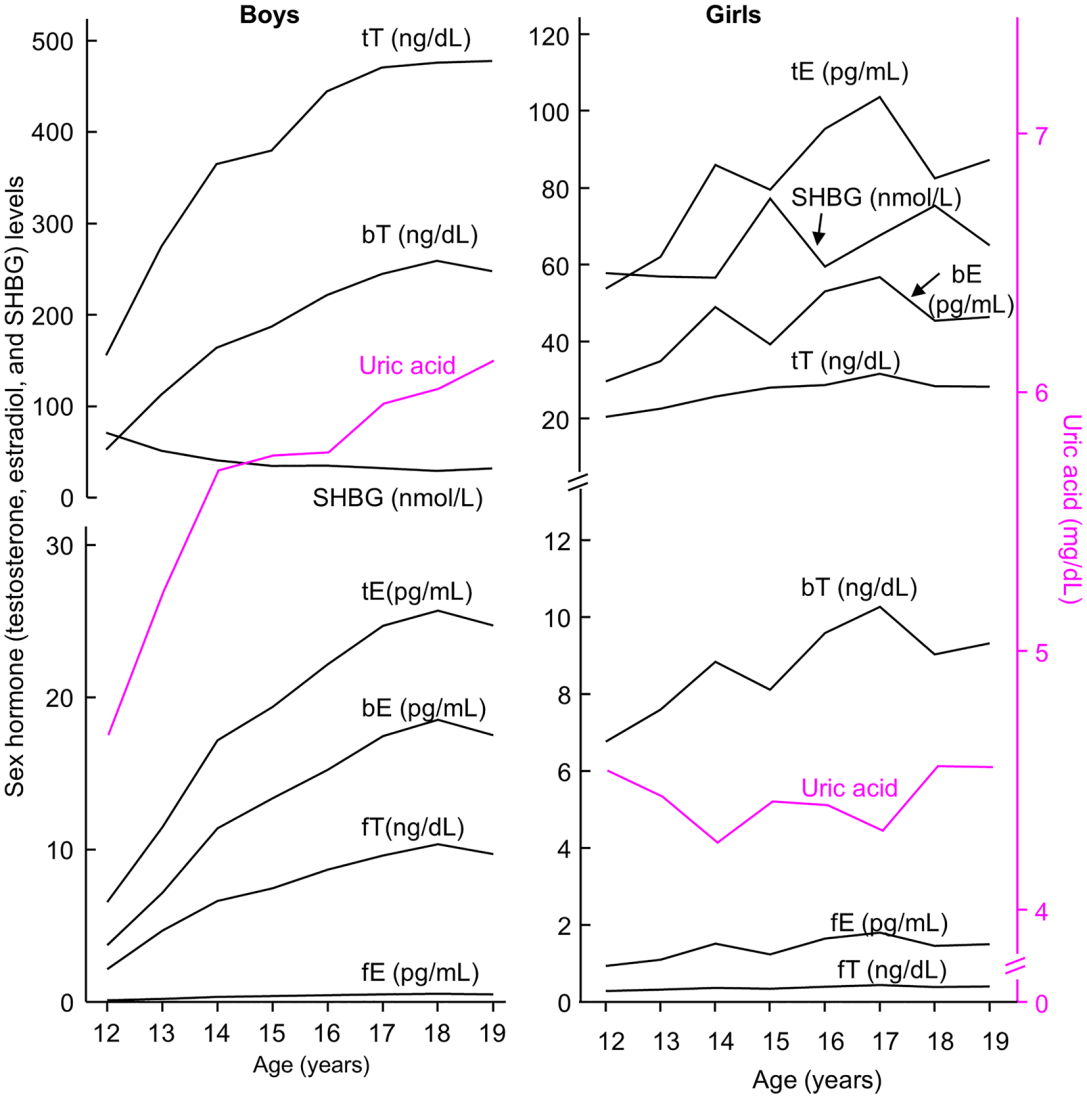
Serum levels of sex hormones and uric acid in adolescents aged 12–19 years. Left panel, 1042 boys which represented a weighted sample size of 13,448,725. Right panel, 993 girls which represented a weighted sample size of 12,291,504. *bE* bioavailable estradiol; *bT* bioavailable testosterone; *fE* free estradiol; *fT* free testosterone; *SHBG* sex hormone-binding globulin; *tE* total estradiol; *tT* total testosterone. Data represent the weighted mean values over each year of age.

**Figure 3:**
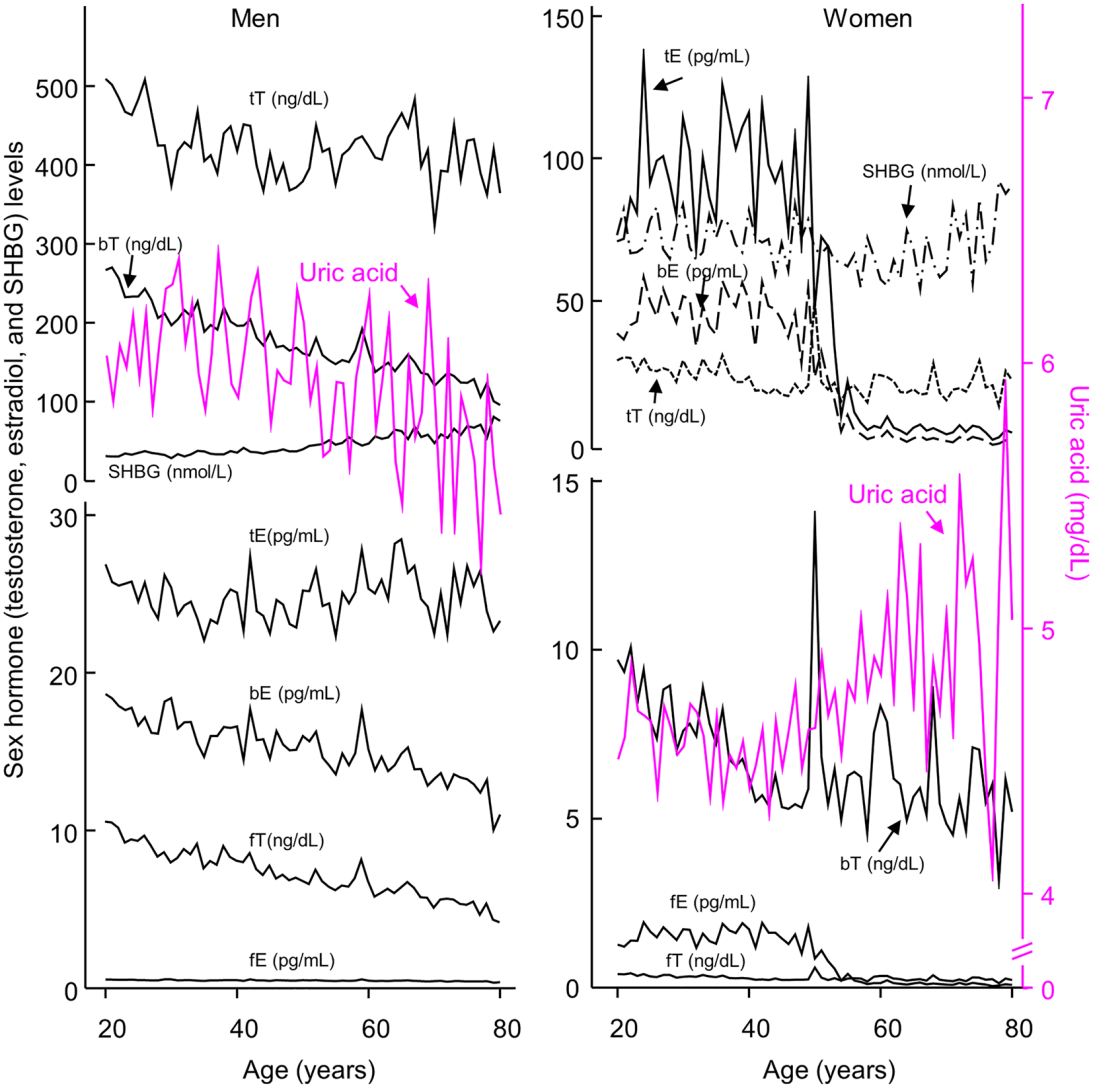
Serum levels of sex hormones and uric acid in adult men and women aged 20–80 years. Left panel, 4037 men which represented a weighted sample size of 88,248,186. Right panel, 3400 women which represented a weighted sample size of 70,353,795. *bE* bioavailable estradiol; *bT* bioavailable testosterone; *fE* free estradiol; *fT* free testosterone; SHBG, sex hormone-binding globulin; *tE* total estradiol; *tT* total testosterone. Data represent the weighted mean values over each year of age.

Total, free, and bioavailable estradiol and testosterone increased during adolescence and then decreased during adulthood in both males and females, except that total estradiol remained relatively unchanged during adulthood in men (Figs. 2 and 3 and Table 2). Sex hormone-binding globulin (SHBG) in males decreased during adolescence and then increased during adulthood; whereas its level in females increased during adolescence and remained relatively unchanged during adulthood (Figs. 2 and 3 and Table 2).

### Establishment of sex difference in circulating uric acid is associated with higher testosterone and lower SHBG in adolescent boys

The establishment of sex difference in uric acid during adolescence coincided with an increase in estradiol in girls without a significant change in uric acid levels in girls during this period (Fig. 2 and Table 2). During the establishment of the sex difference in uric acid, estradiol was not inversely associated with uric acid in either boys or girls (Table 3). Instead, all three fractions of estradiol (free, bioavailable, and total) were positively associated with uric acid in adolescent boys and girls after adjustment for all tested confounders.

**Table 3:** Associations of log-transformed estradiol, testosterone, and sex hormone-binding globulin (independent variable) with uric acid (dependent variable) analyzed by weighted least squares regression.

	Model 1^a^				Model 2^b^			
	Males		Females		Males		Females	
	β	*P* value	β	*P* value	β	*P* value	β	*P* value
**Overall (12–80 years)**								
Log tE	0.079	< 0.001	− 0.062	< 0.001	0.079	< 0.001	− 0.050	0.002
Log fE	0.123	< 0.001	− 0.024	0.148	0.129	< 0.001	− 0.013	0.439
Log bE	0.136	< 0.001	− 0.018	0.274	0.142	< 0.001	− 0.007	0.683
Log tT	− 0.022	0.109	0.020	0.148	− 0.024	0.085	0.028	0.046
Log fT	0.038	0.007	0.128	< 0.001	0.043	0.003	0.132	< 0.001
Log bT	0.049	< 0.001	0.134	< 0.001	0.054	< 0.001	0.138	< 0.001
Log SHBG	− 0.165	< 0.001	− 0.186	< 0.001	− 0.171	< 0.001	− 0.181	< 0.001
**Adolescents (12–19 years)**								
Log tE	0.268	< 0.001	0.092	0.001	0.253	< 0.001	0.091	0.002
Log fE	0.315	< 0.001	0.129	< 0.001	0.301	< 0.001	0.127	< 0.001
Log bE	0.328	< 0.001	0.132	< 0.001	0.314	< 0.001	0.130	< 0.001
Log tT	0.204	< 0.001	0.024	0.408	0.195	< 0.001	0.022	0.451
Log fT	0.258	< 0.001	0.165	< 0.001	0.250	< 0.001	0.159	< 0.001
Log bT	0.267	< 0.001	0.167	< 0.001	0.258	< 0.001	0.161	< 0.001
Log SHBG	− 0.249	< 0.001	− 0.244	< 0.001	− 0.246	< 0.001	− 0.236	< 0.001
**Adults (20–80 years)**								
Log tE	0.043	0.003	− 0.073	< 0.001	0.037	0.011	− 0.070	< 0.001
Log fE	0.090	< 0.001	− 0.037	0.055	0.087	< 0.001	− 0.035	0.071
Log bE	0.106	< 0.001	− 0.031	0.111	0.102	< 0.001	− 0.028	0.139
Log tT	− 0.099	< 0.001	0.030	0.056	− 0.105	< 0.001	0.035	0.027
Log fT	− 0.036	0.029	0.129	< 0.001	− 0.038	0.020	0.131	< 0.001
Log bT	− 0.020	0.240	0.135	< 0.001	− 0.023	0.170	0.137	< 0.001
Log SHBG	− 0.152	< 0.001	− 0.171	< 0.001	− 0.160	< 0.001	− 0.168	< 0.001

*bE, fE or tE* bioavailable, free or total estradiol; *bT, fT or tT* bioavailable, free or total testosterone; *SHBG* sex hormone-binding globulin.

^a^Model 1: adjusted for age, ethnicity, body mass index (BMI, log-transformed), estimated glomerular filtration rate (eGFR), and self-reported health status, lifestyle confounders including smoking status, alcohol consumption, and physical activity.

^b^Model 2: adjusted for all variables in Model 1 plus self-reported comorbidities including hypertension, diabetes, hypercholesterolemia, coronary heart disease, stroke, sleep disorder, gout, and cancer.

This establishment of sex difference in uric acid also coincided with a dramatic increase in testosterone and a decrease in SHBG in boys (Fig. 2). All three fractions of testosterone (log-transformed) were positively associated with serum uric acid in adolescent boys (β = 0.195, *P* < 0.001, for total testosterone; β = 0.250, *P* < 0.001, for free testosterone; and β = 0.258, *P* < 0.001, for bioavailable testosterone; Table 3) after full adjustment. In addition, SHBG (log-transformed) was inversely associated with uric acid in boys (β = − 0.246, *P* < 0.001, Table 3) after full adjustment.

### Association of sex hormones with circulating uric acid in adult men and women

In adult men, all three fractions of estradiol were positively associated with uric acid (Table 3). In this sub-cohort, total testosterone and free testosterone were inversely associated with uric acid, whereas there lacked an association between bioavailable testosterone with uric acid (Table 3).

In adult women and perimenopausal women (aged 47–56 years), free and bioavailable estradiol were not associated with uric acid after full adjustment; however, total estradiol was inversely associated with uric acid in both sub-cohorts after full adjustment (Tables 3 and 4). All three fractions of testosterone were positively associated with uric acid in adult women after full adjustment and so were free and bioavailable testosterone in perimenopausal women (Table 4). Total testosterone was not associated with uric acid in perimenopausal women (Table 4).

**Table 4:** Association of log-transformed sex hormones (independent variables) with uric acid (dependent variable) in 589 females (weighted sample size = 12,736,818) aged 47–56 years analyzed by weighted least squares regression.

	Model 1^a^		Model 2^b^	
	β	*P* value	β	*P* value
Log tE	− 0.109	0.008	− 0.108	0.009
Log fE	− 0.084	0.043	− 0.081	0.052
Log bE	− 0.080	0.056	− 0.076	0.068
Log tT	0.033	0.376	0.040	0.289
Log fT	0.130	< 0.001	0.143	< 0.001
Log bT	0.137	< 0.001	0.150	< 0.001
Log SHBG	− 0.176	< 0.001	− 0.194	< 0.001

*bE, fE or tE* bioavailable, free or total estradiol, *bT, fT or tT* bioavailable, free or total testosterone; *SHBG* sex hormone-binding globulin.

^a^Model 1: adjusted for age, ethnicity, body mass index (BMI, log-transformed), estimated glomerular filtration rate (eGFR), and self-reported health status plus lifestyle confounders including smoking status, alcohol consumption, and physical activity.

^b^Model 2: adjusted for all variables in Model 1 plus self-reported comorbidities including hypertension, diabetes, hypercholesterolemia, coronary heart disease, stroke, sleep disorder, gout, and cancer.

SHBG (log-transformed) remained inversely associated with uric acid in adult men (β = − 0.160, *P* < 0.001) and women (β = − 0.168, *P* < 0.001, Table 3) and perimenopausal women (β = − 0.194, *P* < 0.001, Table 4) after full adjustment.

## Discussion

Using data from a large representative US cohort, this study found that neither free nor bioavailable estradiol was inversely associated with circulating uric acid in adolescents or adults from a general US population after full adjustment. It also found that the sex difference in uric acid was established during adolescence as a result of a dramatic increase in uric acid in adolescent boys. The establishment of sex difference in circulating uric acid was associated with higher testosterone and lower SHBG in adolescent boys.

It is well known that the level of circulating uric acid in males is higher than that in females^20–23^. Experts in the field suspect that this physiological difference may be due to the suppressive effect of estradiol^20,21,25^, because estradiol can inhibit isolated xanthine oxidase (uric acid-generating enzyme)^24^ and it can also pharmacologically decrease circulating uric acid^25^. Our results did not support this speculation. Firstly, during adolescence (12–19 years) when the sex difference in circulating uric acid was established, the significant increase in estradiol over time in girls was accompanied by a relatively unchanged circulating level of uric acid. The establishment of sex difference in uric acid was a result of a dramatic increase in uric acid in adolescent boys. Secondly, when sex difference in uric acid was established during adolescence, estradiol was not inversely associated with uric acid in either boys or girls. Instead, all three fractions of estradiol (free, bioavailable, and total) were positively associated with uric acid in adolescent boys and girls after full adjustment. These results indicate that the establishment of sex difference in uric acid was not a result of the suppressing effect of estradiol on uric acid.

On the other hand, during adolescence when sex difference in uric acid was established, all three fractions of testosterone were positively associated with serum uric acid in adolescent boys. These observations are in agreement with literature reports: testosterone has been reported to stimulate isolated xanthine oxidase^26^, and it can also increase circulating uric acid pharmacologically^25,27^. All these results suggest that testosterone may contribute to the establishment of sex difference in circulating uric acid. However, reversal causality cannot be ruled out.

We also found that SHBG was inversely associated with uric acid in adolescent boys and girls. This result is consistent with a study with a small sample size (N = 205) of diabetic men^28^ that reported a similar finding. The mechanism underlying the observed negative association was not clear. This could be due to that SHBG binds to testosterone thus decreasing the levels of free and bioavailable testosterone. It could also be due to some unknown mechanisms. The establishment of sex difference in circulating uric acid coincided with a significant decrease in SHBG in boys, suggesting that SHBG may play a role in the establishment of such a sex difference. On the other hand, the possibility that uric acid affects the homeostasis of SHBG cannot be ruled out.

Associations of sex hormones with uric acid are more complex and less clear in adult men. For example, uric acid levels decreased slowly and gradually over decades of life in this sub-cohort. Total and free testosterone were inversely associated with uric acid, whereas there lacked an association between bioavailable testosterone with uric acid. These observations contradict the results from adolescent boys where all three types of testosterone were positively associated with uric acid. It is worthwhile to note that the gradual decline in total and free testosterone over decades of adult life was not companied by a gradual increase in uric acid in adult men over time, suggesting that testosterone was less likely to contribute to the gradual decrease in uric acid in men over decades of adult life.

In adult men, all three fractions of estradiol, similar to the findings in adolescent boys and girls, were positively associated with uric acid, suggesting that estradiol was less likely to suppress uric acid in adult men under physiological conditions. The gradual decline in uric acid over time in adult men was companied by a gradual decrease in free and bioavailable estradiol (but not total estradiol), suggesting that lower levels of free and bioavailable estradiol might lead to lower uric acid in adult men, although available reports are against this speculation^24,25^. Again, the possibility that lower uric acid leads to lower estradiol cannot be ruled out in adult men.

The gradual decline in uric acid over time in adult men was also companied by a gradual increase in SHBG, and SHBG was inversely associated with uric acid in this sub-cohort. These findings suggest that SHBG may play a role in regulating uric acid in adult men.

Similar to the situation in men, associations of sex hormones with uric acid are more complex and less clear in adult women. For example, total estradiol, but not free nor bioavailable estradiol, was inversely associated with uric acid in adult women or perimenopausal women after full adjustment. The gradual decrease in estradiol during perimenopause was accompanied by an increase in uric acid, suggesting that total estradiol may suppress uric acid in adult women and contribute to the rise in uric acid later in women’s life. However, it is unknown why there lacked a significant association between biologically active fractions of estradiol and uric acid in adult women, and why this possible suppressing effect of total estradiol on uric acid was only presented in adult women but not adult men nor adolescents.

Consistent with results from adolescents, all three fractions of testosterone in adult women were positively associated with uric acid and so were free and bioavailable testosterone in perimenopausal women, but total testosterone was not associated with uric acid in the latter sub-cohort. In addition, the gradual decrease in testosterone in adult women from their twenties to early forties was companies by a gradual decrease in uric acid during this period, and the increase in uric acid in perimenopausal women seemed accompanied by an overall increase in bioavailable testosterone. These results suggest that bioavailable testosterone may increase uric acid in adult women.

High circulating uric acid, high testosterone, and low SHBG are all associated with some health problems, *e.g*. hypertension^11,29,30^, diabetes^12,30,31^, and cancer^18,30,32^. Whether uric acid is a mediator linking high testosterone- or low SHBG with certain diseases needs to be investigated in the future.

A strength of this study is the large sample size which is representative of the US population. Another strength is that the associations of sex hormones with serum uric acid were adjusted for a wide range of known or perceived confounders. One weakness of this study is that it is based on cross-sectional data; therefore, any causal relationship between testosterone or SHBG and uric acid cannot be established. Another weakness is that data on luteinizing hormone and follicle-stimulating hormone were not available.

In conclusion, in contrast to the experts’ speculation, biologically active estradiol (free and bioavailable estradiol) was not inversely associated with uric acid in adolescents nor adults from a general US population. The sex difference in circulating uric acid was established during adolescence due to a dramatic increase in uric acid in boys and this establishment was associated with higher testosterone and lower SHBG in adolescent boys.

## Methods

### Study participants

NHANES provides data from a representative sample of noninstitutionalized US population. From 2013 to 2016, a total of 13,104 participants had total estradiol, total testosterone, and SHBG data. The following 3632 participants were sequentially excluded: those without albumin (N = 1697) or uric acid (N = 3) or creatinine (N = 1) or body mass index data (N = 115), those pregnant (N = 57), those undergoing hysterectomy or ovary removal (N = 966), those taking sex hormone medication including testosterone, progesterone, estrogen, or unspecified sex hormones (N = 170), and those with kidney disease (estimated glomerular filtration rate < 60 mL/min per 1.73 m^2^, N = 623). Albumin data were needed to calculate free and bioavailable estradiol or testosterone concentrations^33^ and creatinine data were needed to calculate estimated glomerular filtration rate^34^. The resulting 9472 subjects were included in the final analysis of the current study.

### Free and bioavailable estradiol and testosterone

Serum sex hormones were measured at the National Center for Environmental Health. SHBG was quantified based on the reaction of SHBG with immuno-antibodies and chemo-luminescence measurements of the reaction products by a photomultiplier tube^35^. Total testosterone and estradiol were measured using an isotope dilution-high performance liquid chromatography-tandem mass spectrometry^36^.

Estradiol and testosterone mainly exist in three fractions: free, albumin-bound, or SHBG bound. The fraction that is bound to SHBG is biologically inactive. Bioavailable estradiol or testosterone is the biologically active fraction of estradiol or testosterone, and it includes both free and albumin-bound fractions of each hormone. Free and bioavailable estradiol and testosterone were calculated according to the method provided by Vermeulen and colleagues^33^ based on serum concentrations of total estradiol or testosterone, SHBG, and albumin.

### Uric acid

Uric acid was measured using the uricase peroxidase method^37^. In brief, uric acid was oxidized by uricase to produce hydrogen peroxide and the latter produced a colored product in the presence of peroxidase. The absorbance of the colored product, being measured at 520 nm, was directly proportional to the concentration of uric acid in the sample.

### eGFR calculation and definition of kidney disease

eGFR was calculated by the CKD-EPI equation^34^: eGFR = 141 × min(Scr/κ, 1)^α^ × max(Scr/κ, 1)^− 1.209^ × 0.993^Age^ × 1.018 [if female] × 1.159 [if black], where Scr is serum creatinine, κ is 0.7 for females and 0.9 for males, α is − 0.329 for females and − 0.411 for males, min indicates the minimum of Scr/κ or 1, and max indicates the maximum of Scr/κ or 1. Kidney disease^38^ was defined as eGFR < 60 mL/min per 1.73 m^2^ and people with kidney disease were excluded from this study because impaired kidney function significantly increases uric acid levels in the circulation^6,10^.

### Covariates

Confounding covariates included age, ethnicity (Hispanic, non-Hispanic white, non-Hispanic black, or other), body mass index (continuous variable), eGFR (continuous variable, an indicator of kidney function), and self-reported health status (excellent, very good, good, fair, poor, or unknown). Lifestyle confounders included smoking status (never, former, current, or unknown), alcohol consumption (non-drinker, former drinker, current drinker, or unknown), and leisure-time physical activity (0, 1–149, 150–299, and ≥ 300 min per week, or unknown)^39,40^. Leisure-time physical activity was calculated using the following formula^40^: leisure-time physical activity (min/week) = 2 × vigorous activity frequency × vigorous activity duration + moderate activity frequency × moderate activity duration. Self-reported comorbidities included self-reported diagnoses of hypertension (yes, no, or unknown), diabetes (yes, no, borderline, or unknown), hypercholesterolemia (yes, no, or unknown), coronary heart disease (yes, no, or unknown), stroke (yes, no, or unknown), sleep disorder (yes, no, or unknown), gout (yes, no, or unknown), and cancer (yes, no, or unknown).

### Statistical analysis

Data from the two NHANES cycles (2013–2014 and 2015–2016) were combined using the appropriate weighting methods^41^. Four-year weights were calculated by dividing the two-year weights by two^42^ and used in all analyses to adjust for unequal selection probability and non-response bias following NHANES analytical guidelines^41^. Population means, medians, and proportions were estimated and reported. Uric acid, age, and eGFR were approximately normally distributed. Estradiol, testosterone, SHBG, and body mass index were not normally distributed, and these variables were log-transformed before regression analyses were conducted. Descriptive statistics were presented as median and interquartile range (non-normally distributed continuous data), or mean and SD (approximately normally distributed continuous data), or percentages (categorical data). The association analyses were conducted using weighted least squares regression^43^ with or without adjusting for covariates. Sub-analyses were conducted in the following sub-cohorts: participants aged 12–19 years or 20–80 years or perimenopausal women aged 47–56 years. All tests were two-sided and a *P* value of < 0.05 was regarded as statistically significant. All statistical analyses were performed using SPSS version 27.0 (IBM SPSS Statistics for Windows, Armonk, NY, International Business Machines Corporation).

### Ethical considerations

The National Center for Health Statistics Research Ethics Review Board (ERB) approved all study protocols (ERB No. NHANES Protocol #2011-17). All research procedures were performed following the relevant guidelines and regulations. Written informed consent was obtained from all participants. For participants who were under 18 years old and were 12 years old or older (the youngest age of this study was 12 years), written informed consent was obtained from both the participant and a parent or guardian of the participant; for emancipated minors who were 16 or 17 years old, the written informed consent was obtained from the participant only. The participants’ records were anonymized before being accessed by the author.

## Supplementary Information

Supplementary Information.

## Data Availability

All data in the current analysis are publicly available on the NHANES website.
