# Supplementary material for: Establishment of sex difference in circulating uric acid is associated with higher testosterone and lower sex hormone-binding globulin in adolescent boys
Source: Sci Rep. 2021 Aug 30;11:17323. doi: 10.1038/s41598-021-96959-4 (PMC8405811; doi:10.1038/s41598-021-96959-4)
Supplement: Supplementary file 1 — Supplementary Information. [file 41598_2021_96959_MOESM1_ESM.pdf]

Supplementary Information

Title: Establishment of sex difference in circulating uric acid is associated with higher testosterone and lower sex hormone-binding globulin in adolescent boys

Authors: Yutang Wang, PhD, Fadi J Charchar, PhD

Supplementary Table 1: Characteristics of the whole cohort

	Overall	Males	Females
No., unweighted	9 472	5 079	4 393
No., weighted	184 342 210	101 696 911	82 645 299
tE, median (IQR), pg/mL	24.9 (16.4-40.6)	23.1 (17.8-28.7)	40.5 (9.5-102.0)
fE, median (IQR), pg/mL	0.5 (0.3-0.8)	0.5 (0.3-0.6)	0.7 (0.2-1.6)
bE, median (IQR), pg/mL	15.6 (9.8-24.6)	14.6 (11.0-18.6)	21.8 (5.4-49.6)
tT, median (IQR), ng/dL	210.0 (23.1-415.0)	394.0 (294.0-516.7)	21.8 (15.6-30.4)
fT, median (IQR), ng/dL	4.1 (0.3-7.5)	7.1 (5.5-9.2)	0.3 (0.2-0.4)
bT, median (IQR), ng/dL	95.7 (6.4-180.8)	172.3 (131.0-224.5)	5.9 (3.9-9.0)
SHBG, median (IQR), nmol/L	45.0 (30.5-68.2)	36.8 (26.2-52.2)	59.5 (40.1-88.4)
Uric acid, mean (SD), mg/dL	5.4 (1.3)	5.9 (1.2)	4.6 (1.1)
Age, mean (SD), y	40.7 (17.9)	41.6 (18.1)	39.5 (17.5)
BMI, median (IQR), kg/m^2	27.3 (23.4-32.1)	27.5 (24.0-31.6)	27.1 (22.8-32.8)
eGFR, mean (SD), mL/min per 1.73 m^2	103.8 (22.2)	102.2 (22.5)	105.8 (21.5)
Ethnicity, %			
Hispanic	17.8	17.2	18.6
Non-Hispanic white	61.8	63.5	59.8
Non-Hispanic black	11.0	10.2	11.9
Other	9.4	9.1	9.7
Health status, %			
Excellent	10.1	10.0	10.3
Very good	30.6	29.9	31.4
Good	37.2	39.9	33.9
Fair	13.1	13.2	13.0
Poor	1.7	1.5	1.9
Unknown	7.3	5.6	9.5
Physical activity, %			
0 min per week	40.4	39.3	41.7
1-149 min per week	15.3	13.9	17.0
150-299 min per week	12.8	12.3	13.5
≥300 min per week	31.2	34.2	27.5
Unknown	0.3	0.3	0.3
Smoking status, %			

Never	51.5	46.2	58.0
Former	20.4	24.8	14.9
Current	17.2	18.7	15.2
Unknown	11.0	10.2	11.8
Alcohol consumption, %			
Non-drinker	10.8	7.8	14.6
Former drinker	7.7	5.3	10.6
Current drinker	63.4	71.3	53.7
Unknown	18.1	15.7	21.1
Hypertension, %	24.3	26.4	21.8
Diabetes, %	7.5	8.8	6.0
Hypercholesterolemia, %	26.5	30.4	21.7
CHD, %	2.0	2.9	0.8
Stroke, %	1.5	1.7	1.3
Gout, %	2.5	3.9	0.8
Sleep disorder, %	23.2	21.9	24.8
Cancer, %	6.6	7.3	5.7

bE, fE or tE: bioavailable, free or total estradiol; BMI: body mass index; bT, fT or tT: bioavailable, free or total testosterone; CHD: coronary heart disease; eGFR: estimated glomerular filtration rate; IQR: interquartile range; No.: number; SD: standard deviation; SHBG: sex hormone-binding globulin.
